# 1-(4-Ferrocenylphen­yl)-3-methyl­imidazolium iodide monohydrate

**DOI:** 10.1107/S1600536809051216

**Published:** 2009-12-12

**Authors:** Douglas Onyancha, Cedric McCleland, Thomas Gerber, Eric Hosten, Peter Mayer

**Affiliations:** a Department of Chemistry, Nelson Mandela Metropolitan University, 6031 Port Elizabeth, South Africa; b Department of Chemistry, Ludwig-Maximilians University, D-81377 München, Germany

## Abstract

In the title compound, [Fe(C_5_H_5_)(C_15_H_14_N_2_)]I·H_2_O, the benzene and imidazolium rings are twisted by 17.26 (17) and 32.53 (19)°, respectively, with respect to the η^5^-C_5_H_4_ plane of the ferrocenyl unit. The imidazolium ring is rotated by 48.81 (17)° with respect to the benzene ring. The packing is dominated by layers established by O—H⋯I, C—H⋯I and C—H⋯O contacts and propagating along the *bc* plane.

## Related literature

For imidazolium salts see: Nolan *et al.* (2007 ▶); Cheng *et al.* (2008 ▶); Yang *et al.* (2009 ▶); Bildstein *et al.* (1999 ▶). For the synthesis, see: Zhao *et al.* (2001 ▶); Koten *et al.* (2007 ▶). For graph-set analysis, see: Bernstein *et al.* (1995 ▶); Etter *et al.* (1990 ▶).
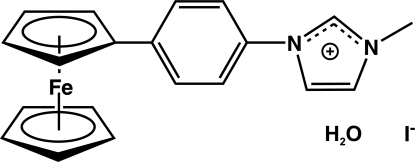


## Experimental

### 

#### Crystal data

[Fe(C_5_H_5_)(C_15_H_14_N_2_)]I·H_2_O
*M* *_r_* = 488.14
Monoclinic, 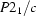

*a* = 17.3871 (7) Å
*b* = 7.3397 (2) Å
*c* = 16.9445 (6) Å
β = 117.299 (5)°
*V* = 1921.56 (14) Å^3^
*Z* = 4
Mo *K*α radiation
μ = 2.40 mm^−1^
*T* = 200 K
0.50 × 0.39 × 0.04 mm

#### Data collection

Oxford Xcalibur diffractometer
Absorption correction: numerical [*X-SHAPE* (Stoe & Cie, 1996 ▶) and *X-RED* (Stoe & Cie, 1997 ▶)] *T*_min_ = 0.399, *T*_max_ = 0.901
12113 measured reflections
3885 independent reflections
3098 reflections with *I* > 2σ(*I*)
*R*_int_ = 0.042

#### Refinement

*R*[*F* ^2^ > 2σ(*F* ^2^)] = 0.032
*wR*(*F* ^2^) = 0.081
*S* = 0.97
3885 reflections
227 parameters
H-atom parameters constrained
Δρ_max_ = 1.28 e Å^−3^
Δρ_min_ = −0.50 e Å^−3^

### 

Data collection: *CrysAlis PRO* (Oxford Diffraction, 2006 ▶); cell refinement: *CrysAlis PRO*; data reduction: *CrysAlis PRO*; program(s) used to solve structure: *SHELXS97* (Sheldrick, 2008 ▶); program(s) used to refine structure: *SHELXL97* (Sheldrick, 2008 ▶); molecular graphics: *ORTEP-3 for Windows* (Farrugia, 1997 ▶) and *Mercury* (Macrae *et al.*, 2006 ▶); software used to prepare material for publication: *publCIF* (Westrip, 2009 ▶).

## Supplementary Material

Crystal structure: contains datablocks I, global. DOI: 10.1107/S1600536809051216/ng2694sup1.cif

Structure factors: contains datablocks I. DOI: 10.1107/S1600536809051216/ng2694Isup2.hkl

Additional supplementary materials:  crystallographic information; 3D view; checkCIF report

## Figures and Tables

**Table 1:** Hydrogen-bond geometry (Å, °)

*D*—H⋯*A*	*D*—H	H⋯*A*	*D*⋯*A*	*D*—H⋯*A*
O1—H1*A*⋯I1	0.84	2.74	3.575 (3)	176
O1—H1*B*⋯I1^i^	0.84	2.78	3.616 (3)	176
C12—H12*A*⋯O1	0.95	2.39	3.277 (4)	156
C13—H13⋯I1^ii^	0.95	3.01	3.931 (3)	163

Symmetry codes: (i) 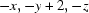; (ii) 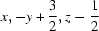.

**Fig. 1.**
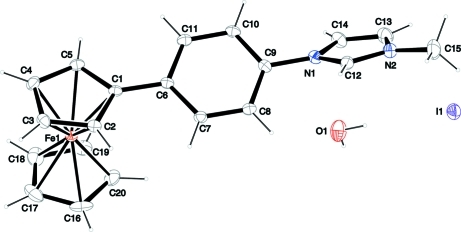
The molecular structure of the title compound (anisotropic displacement ellipsoids drawn at the 50% probability level).

**Fig. 2.**
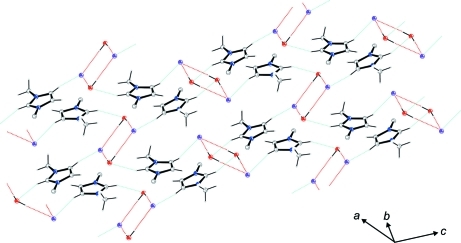
two-dimensional layer propagating along the bc plane. Red dotted lines indicate O–H···I contacts, blue dotted lines indicate C–H···O and C–H···I contacts. Ferrocenyl-C5H4 moieties have been omitted for clarity.

**Fig. 3.**
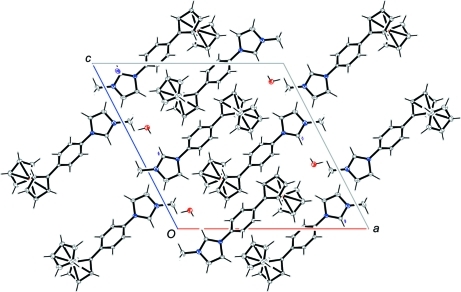
The packing of the title compound viewed along [010].

## supplementary crystallographic information

### Comment

The structure consists of a ferrocenyl group attached to a phenyl-imidazolium moiety in *para*-position, an iodide anion and a water molecule (Fig. 1). The phenyl ring A is twisted by 17.26 (17)° away from the least-square plane B of the cyclopentadienyl ring, and by 48.81 (17)° from the imidazole ring C. The plane of the imidazole unit C is rotated by 32.53 (19)° with respect to the η^5^-C_5_H_4_ plane A (for plane definition, see Table 2).

The packing of the title compound is dominated by two-dimensional layers parallel to the *bc* plane formed by O–H···I, C–H···I and C–H···O contacts (Fig. 2). Eight-membered rings consisting of two water molecules and two iodide anions are formed by four O–H···I contacts. According to graph-set theory (Bernstein *et al.* 1995; Etter *et al.* 1990), the descriptor *R*_4_^2^(8) can be assigned on the binary level for these rings. Additionally each of the two oxygen and the two iodide atoms of the rings acts as an acceptor in C–H···O/I contacts to four different imidazole moieties.

The packing of the title compound viewed along [010] is shown in Figure 3.

### Experimental

All chemicals used (reagent grade) were commercially available. 4-Bromophenylferrocene (Zhao *et al.* 2001) and 4-ferrocenylphenyl-1*H*-imidazole (Koten *et al.* 2007) were prepared according to literature methods. In a round- bottomed flask, iodomethane (5 ml) was added to 4-ferrocenylphenyl-1*H*-imidzole and allowed to reflux gently under a nitrogen atmosphere for 3 h. The reaction was monitored visually, and the yellow colour of the solution gradually turned colourless, signaling the completion of the reaction. After cooling, the yellow precipitate was filtered off under vacuum and washed with ether (yield, 99%). Recrystallization was from a dichloromethane/toluene mixture. *M*.p. 130–132°C, IR (KBr cm^-1^): 3463, 3403, 3080, 1616, 1554, 1455, 1262, 1222, 1003, 887, 823, 751; ^1^H NMR (300 MHz, CDCl_3_); 10.64 (1*H*, s, ImH), 7.65–7.69 (6*H*, m, 4×ArH, 2×ImH), 4.69 (2*H*, t, J 1.9, C_5_H_4_), 4.42 (2H. t, J 1.9, C_5_H_4_), 4.28 (3*H*, s, CH_3_), 4.07 (5*H*, s, C_5_H_5_); ^13^C NMR 134.02, 135.70, 131.60, 127.64, 124.19, 122.07, 120.48, 82.39, 69.94, 69.88, 66.81, 37.66.

### Refinement

The C-bound H atoms are riding on their parent atoms (C—H = 0.98 Å for CH_3_, 0.95 Å for CH) with *U*_iso_(H) = 1.2*U*_eq_(C) for CH, *U*_iso_(H) = 1.5*U*_eq_(C) for CH_3_]. The O-bound H are riding on their parent atom (O—H = 0.84 Å) with *U*_iso_(H) = 1.5*U*_eq_(O).

### Crystal data

**Table:**

[Fe(C_5_H_5_)(C_15_H_14_N_2_)]I·H_2_O	*F*(000) = 968
*M**_r_* = 488.14	*D*_x_ = 1.687 (1) Mg m^−^^3^
Monoclinic, *P*2_1_/*c*	Mo *K*α radiation, λ = 0.71073 Å
Hall symbol: -P 2ybc	Cell parameters from 6938 reflections
*a* = 17.3871 (7) Å	θ = 4.4–26.3°
*b* = 7.3397 (2) Å	µ = 2.40 mm^−^^1^
*c* = 16.9445 (6) Å	*T* = 200 K
β = 117.299 (5)°	Platelet, orange
*V* = 1921.56 (14) Å^3^	0.50 × 0.39 × 0.04 mm
*Z* = 4	

### Data collection

**Table:**

Oxford Xcalibur diffractometer	3885 independent reflections
Radiation source: fine-focus sealed tube	3098 reflections with *I* > 2σ(*I*)
graphite	*R*_int_ = 0.042
Detector resolution: 15.9809 pixels mm^-1^	θ_max_ = 26.4°, θ_min_ = 4.5°
ω scans	*h* = −21→19
Absorption correction: numerical [*X-SHAPE* (Stoe & Cie, 1996) and *X-RED* (Stoe & Cie, 1997)]	*k* = −9→9
*T*_min_ = 0.399, *T*_max_ = 0.901	*l* = −16→21
12113 measured reflections	

### Refinement

**Table:**

Refinement on *F*^2^	Primary atom site location: structure-invariant direct methods
Least-squares matrix: full	Secondary atom site location: difference Fourier map
*R*[*F*^2^ > 2σ(*F*^2^)] = 0.032	Hydrogen site location: inferred from neighbouring sites
*wR*(*F*^2^) = 0.081	H-atom parameters constrained
*S* = 0.97	*w* = 1/[σ^2^(*F*_o_^2^) + (0.049*P*)^2^] where *P* = (*F*_o_^2^ + 2*F*_c_^2^)/3
3885 reflections	(Δ/σ)_max_ = 0.001
227 parameters	Δρ_max_ = 1.28 e Å^−^^3^
0 restraints	Δρ_min_ = −0.50 e Å^−^^3^

### Special details

**Table:**

Experimental. Crystal faces optimized with XShape, Version 1.02 (Stoe, 1996) Absorption correction with XRed, Version 1.09 (Stoe, 1997)
Refinement. The C-bound H atoms are riding on their parent atoms (C—H = 0.98 Å for CH_3_, 0.95 Å for CH) with *U*_iso_(H) = 1.2*U*_eq_(C) for CH, *U*_iso_(H) = 1.5*U*_eq_(C) for CH_3_]. The O-bound H are riding on their parent atom (O—H = 0.84 Å) with *U*_iso_(H) = 1.5*U*_eq_(O).

### Fractional atomic coordinates and isotropic or equivalent isotropic displacement parameters (Å^2^)

**Table:**

	*x*	*y*	*z*	*U*_iso_*/*U*_eq_	
Fe1	0.63876 (3)	0.84486 (5)	0.20164 (3)	0.02466 (12)	
N1	0.18768 (18)	0.6660 (3)	−0.07900 (16)	0.0282 (6)	
N2	0.04809 (19)	0.6670 (3)	−0.13933 (19)	0.0364 (7)	
C1	0.5395 (2)	0.6743 (4)	0.18554 (19)	0.0235 (6)	
C2	0.5720 (2)	0.7706 (4)	0.26899 (19)	0.0263 (6)	
H2	0.5395	0.8488	0.2870	0.032*	
C3	0.6614 (2)	0.7277 (4)	0.3194 (2)	0.0320 (7)	
H3	0.6989	0.7726	0.3770	0.038*	
C4	0.6848 (2)	0.6060 (4)	0.2687 (2)	0.0330 (7)	
H4	0.7406	0.5555	0.2864	0.040*	
C5	0.6103 (2)	0.5735 (4)	0.1875 (2)	0.0287 (7)	
H5	0.6078	0.4964	0.1414	0.034*	
C6	0.4495 (2)	0.6764 (4)	0.11489 (19)	0.0226 (6)	
C7	0.3910 (2)	0.8094 (4)	0.1133 (2)	0.0265 (7)	
H7	0.4107	0.9028	0.1569	0.032*	
C8	0.3054 (2)	0.8078 (4)	0.0495 (2)	0.0279 (7)	
H8	0.2668	0.9000	0.0488	0.034*	
C9	0.2766 (2)	0.6703 (4)	−0.01333 (19)	0.0253 (6)	
C10	0.3324 (2)	0.5365 (4)	−0.01372 (19)	0.0295 (7)	
H10	0.3121	0.4425	−0.0571	0.035*	
C11	0.4182 (2)	0.5411 (4)	0.04971 (19)	0.0288 (7)	
H11A	0.4567	0.4502	0.0490	0.035*	
C12	0.1202 (2)	0.6788 (4)	−0.0630 (2)	0.0295 (7)	
H12A	0.1228	0.6940	−0.0061	0.035*	
C13	0.0705 (3)	0.6437 (5)	−0.2066 (2)	0.0423 (9)	
H13	0.0315	0.6302	−0.2679	0.051*	
C14	0.1569 (2)	0.6435 (5)	−0.1704 (2)	0.0359 (8)	
H14	0.1908	0.6305	−0.2009	0.043*	
C15	−0.0396 (3)	0.6651 (6)	−0.1483 (3)	0.0570 (11)	
H15A	−0.0673	0.5483	−0.1735	0.085*	
H15B	−0.0733	0.7644	−0.1877	0.085*	
H15C	−0.0371	0.6814	−0.0898	0.085*	
C16	0.6389 (3)	1.1210 (5)	0.2001 (3)	0.0510 (11)	
H16	0.6180	1.1974	0.2312	0.061*	
C17	0.7237 (3)	1.0564 (5)	0.2324 (3)	0.0541 (11)	
H17	0.7706	1.0814	0.2891	0.065*	
C18	0.7271 (3)	0.9470 (5)	0.1652 (3)	0.0483 (9)	
H18	0.7766	0.8844	0.1693	0.058*	
C19	0.6455 (2)	0.9471 (4)	0.0923 (2)	0.0375 (8)	
H19	0.6300	0.8862	0.0377	0.045*	
C20	0.5897 (3)	1.0525 (4)	0.1130 (2)	0.0427 (9)	
H20	0.5300	1.0739	0.0755	0.051*	
O1	0.11333 (17)	0.8580 (4)	0.11076 (16)	0.0511 (7)	
H1A	0.0611	0.8425	0.0980	0.077*	
H1B	0.1159	0.9349	0.0753	0.077*	
I1	−0.112089 (15)	0.80931 (3)	0.047195 (15)	0.04024 (10)	

### Atomic displacement parameters (Å^2^)

**Table:**

	*U*^11^	*U*^22^	*U*^33^	*U*^12^	*U*^13^	*U*^23^
Fe1	0.0253 (2)	0.0217 (2)	0.0259 (2)	−0.00069 (16)	0.01074 (19)	0.00191 (17)
N1	0.0316 (15)	0.0281 (14)	0.0238 (13)	−0.0031 (11)	0.0117 (12)	0.0003 (10)
N2	0.0327 (16)	0.0341 (15)	0.0361 (15)	0.0009 (12)	0.0104 (13)	−0.0007 (12)
C1	0.0322 (18)	0.0182 (14)	0.0226 (14)	−0.0026 (11)	0.0146 (13)	0.0007 (11)
C2	0.0317 (18)	0.0247 (15)	0.0251 (15)	−0.0015 (12)	0.0153 (14)	−0.0009 (12)
C3	0.0318 (19)	0.0350 (17)	0.0236 (15)	−0.0044 (14)	0.0079 (14)	0.0038 (13)
C4	0.0300 (18)	0.0261 (16)	0.0416 (18)	0.0066 (13)	0.0154 (16)	0.0080 (14)
C5	0.0363 (18)	0.0218 (15)	0.0309 (16)	−0.0003 (13)	0.0179 (15)	0.0031 (13)
C6	0.0289 (17)	0.0201 (14)	0.0226 (14)	−0.0032 (11)	0.0150 (13)	0.0006 (11)
C7	0.0297 (17)	0.0236 (15)	0.0266 (15)	−0.0003 (12)	0.0132 (14)	−0.0040 (12)
C8	0.0321 (18)	0.0239 (15)	0.0299 (16)	0.0011 (12)	0.0160 (14)	−0.0029 (12)
C9	0.0285 (17)	0.0260 (15)	0.0211 (14)	−0.0048 (12)	0.0111 (13)	0.0007 (11)
C10	0.0374 (19)	0.0261 (16)	0.0259 (15)	−0.0031 (13)	0.0153 (14)	−0.0081 (12)
C11	0.0350 (18)	0.0241 (15)	0.0317 (16)	0.0008 (13)	0.0190 (15)	−0.0036 (12)
C12	0.0296 (18)	0.0301 (18)	0.0273 (16)	0.0016 (12)	0.0118 (14)	−0.0020 (12)
C13	0.046 (2)	0.045 (2)	0.0262 (17)	−0.0032 (16)	0.0088 (17)	−0.0016 (15)
C14	0.040 (2)	0.0430 (19)	0.0221 (15)	−0.0068 (15)	0.0123 (15)	−0.0038 (14)
C15	0.029 (2)	0.069 (3)	0.063 (3)	0.0035 (18)	0.013 (2)	−0.008 (2)
C16	0.089 (4)	0.0171 (16)	0.062 (3)	−0.0045 (18)	0.048 (3)	−0.0013 (16)
C17	0.058 (3)	0.049 (2)	0.045 (2)	−0.028 (2)	0.015 (2)	−0.0007 (18)
C18	0.041 (2)	0.051 (2)	0.057 (2)	−0.0043 (17)	0.025 (2)	0.0150 (19)
C19	0.051 (2)	0.0315 (18)	0.0346 (18)	−0.0023 (15)	0.0230 (17)	0.0072 (14)
C20	0.044 (2)	0.0325 (18)	0.046 (2)	0.0065 (15)	0.0164 (18)	0.0181 (15)
O1	0.0404 (16)	0.0708 (17)	0.0442 (14)	0.0056 (13)	0.0213 (13)	0.0096 (13)
I1	0.03337 (15)	0.03911 (15)	0.03887 (15)	−0.00048 (9)	0.00850 (11)	0.00466 (9)

### Geometric parameters (Å, °)

**Table:**

Fe1—C16	2.027 (3)	C7—C8	1.381 (4)
Fe1—C20	2.032 (3)	C7—H7	0.9500
Fe1—C17	2.039 (4)	C8—C9	1.383 (4)
Fe1—C3	2.039 (3)	C8—H8	0.9500
Fe1—C5	2.040 (3)	C9—C10	1.384 (4)
Fe1—C2	2.040 (3)	C10—C11	1.382 (4)
Fe1—C18	2.042 (4)	C10—H10	0.9500
Fe1—C4	2.042 (3)	C11—H11A	0.9500
Fe1—C1	2.046 (3)	C12—H12A	0.9500
Fe1—C19	2.052 (3)	C13—C14	1.337 (5)
N1—C12	1.323 (4)	C13—H13	0.9500
N1—C14	1.397 (4)	C14—H14	0.9500
N1—C9	1.432 (4)	C15—H15A	0.9800
N2—C12	1.328 (4)	C15—H15B	0.9800
N2—C13	1.374 (5)	C15—H15C	0.9800
N2—C15	1.461 (5)	C16—C17	1.399 (6)
C1—C5	1.424 (4)	C16—C20	1.416 (5)
C1—C2	1.444 (4)	C16—H16	0.9486
C1—C6	1.472 (4)	C17—C18	1.416 (5)
C2—C3	1.423 (4)	C17—H17	0.9503
C2—H2	0.9490	C18—C19	1.390 (5)
C3—C4	1.423 (5)	C18—H18	0.9496
C3—H3	0.9500	C19—C20	1.407 (5)
C4—C5	1.411 (4)	C19—H19	0.9485
C4—H4	0.9496	C20—H20	0.9492
C5—H5	0.9493	O1—H1A	0.8400
C6—C11	1.397 (4)	O1—H1B	0.8400
C6—C7	1.400 (4)		
			
C16—Fe1—C20	40.82 (15)	C3—C4—H4	126.0
C16—Fe1—C17	40.26 (17)	Fe1—C4—H4	126.4
C20—Fe1—C17	68.09 (16)	C4—C5—C1	109.4 (3)
C16—Fe1—C3	115.66 (14)	C4—C5—Fe1	69.87 (17)
C20—Fe1—C3	150.05 (15)	C1—C5—Fe1	69.85 (16)
C17—Fe1—C3	106.21 (14)	C4—C5—H5	125.3
C16—Fe1—C5	167.57 (17)	C1—C5—H5	125.3
C20—Fe1—C5	130.44 (14)	Fe1—C5—H5	126.6
C17—Fe1—C5	151.86 (16)	C11—C6—C7	117.7 (3)
C3—Fe1—C5	68.33 (13)	C11—C6—C1	121.3 (3)
C16—Fe1—C2	106.16 (14)	C7—C6—C1	121.0 (2)
C20—Fe1—C2	117.37 (14)	C8—C7—C6	121.4 (3)
C17—Fe1—C2	126.28 (15)	C8—C7—H7	119.3
C3—Fe1—C2	40.85 (13)	C6—C7—H7	119.3
C5—Fe1—C2	68.62 (12)	C7—C8—C9	119.3 (3)
C16—Fe1—C18	67.90 (17)	C7—C8—H8	120.4
C20—Fe1—C18	67.70 (15)	C9—C8—H8	120.4
C17—Fe1—C18	40.60 (16)	C8—C9—C10	120.9 (3)
C3—Fe1—C18	128.05 (15)	C8—C9—N1	119.7 (3)
C5—Fe1—C18	119.89 (15)	C10—C9—N1	119.4 (3)
C2—Fe1—C18	165.18 (14)	C11—C10—C9	119.2 (3)
C16—Fe1—C4	149.65 (16)	C11—C10—H10	120.4
C20—Fe1—C4	168.37 (14)	C9—C10—H10	120.4
C17—Fe1—C4	117.31 (15)	C10—C11—C6	121.5 (3)
C3—Fe1—C4	40.79 (13)	C10—C11—H11A	119.3
C5—Fe1—C4	40.45 (12)	C6—C11—H11A	119.3
C2—Fe1—C4	68.75 (13)	N1—C12—N2	109.0 (3)
C18—Fe1—C4	109.05 (15)	N1—C12—H12A	125.5
C16—Fe1—C1	127.99 (16)	N2—C12—H12A	125.5
C20—Fe1—C1	108.52 (13)	C14—C13—N2	107.8 (3)
C17—Fe1—C1	165.36 (16)	C14—C13—H13	126.1
C3—Fe1—C1	69.20 (12)	N2—C13—H13	126.1
C5—Fe1—C1	40.79 (12)	C13—C14—N1	106.7 (3)
C2—Fe1—C1	41.39 (11)	C13—C14—H14	126.7
C18—Fe1—C1	152.79 (14)	N1—C14—H14	126.7
C4—Fe1—C1	68.92 (12)	N2—C15—H15A	109.5
C16—Fe1—C19	67.81 (14)	N2—C15—H15B	109.5
C20—Fe1—C19	40.28 (14)	H15A—C15—H15B	109.5
C17—Fe1—C19	67.52 (15)	N2—C15—H15C	109.5
C3—Fe1—C19	166.65 (14)	H15A—C15—H15C	109.5
C5—Fe1—C19	111.23 (13)	H15B—C15—H15C	109.5
C2—Fe1—C19	152.35 (14)	C17—C16—C20	108.1 (3)
C18—Fe1—C19	39.69 (14)	C17—C16—Fe1	70.3 (2)
C4—Fe1—C19	130.26 (13)	C20—C16—Fe1	69.78 (19)
C1—Fe1—C19	119.82 (13)	C17—C16—H16	125.9
C12—N1—C14	108.1 (3)	C20—C16—H16	125.9
C12—N1—C9	125.6 (3)	Fe1—C16—H16	125.5
C14—N1—C9	126.3 (3)	C16—C17—C18	107.7 (4)
C12—N2—C13	108.4 (3)	C16—C17—Fe1	69.4 (2)
C12—N2—C15	125.2 (3)	C18—C17—Fe1	69.8 (2)
C13—N2—C15	126.3 (3)	C16—C17—H17	126.2
C5—C1—C2	106.6 (3)	C18—C17—H17	126.1
C5—C1—C6	127.6 (3)	Fe1—C17—H17	126.2
C2—C1—C6	125.7 (3)	C19—C18—C17	108.2 (4)
C5—C1—Fe1	69.36 (17)	C19—C18—Fe1	70.5 (2)
C2—C1—Fe1	69.07 (17)	C17—C18—Fe1	69.6 (2)
C6—C1—Fe1	128.30 (19)	C19—C18—H18	125.9
C3—C2—C1	108.0 (3)	C17—C18—H18	125.9
C3—C2—Fe1	69.55 (18)	Fe1—C18—H18	125.6
C1—C2—Fe1	69.54 (17)	C18—C19—C20	108.5 (3)
C3—C2—H2	126.0	C18—C19—Fe1	69.76 (19)
C1—C2—H2	126.0	C20—C19—Fe1	69.10 (18)
Fe1—C2—H2	126.5	C18—C19—H19	125.8
C4—C3—C2	108.2 (3)	C20—C19—H19	125.7
C4—C3—Fe1	69.73 (17)	Fe1—C19—H19	126.9
C2—C3—Fe1	69.60 (17)	C19—C20—C16	107.5 (3)
C4—C3—H3	126.0	C19—C20—Fe1	70.61 (19)
C2—C3—H3	125.9	C16—C20—Fe1	69.39 (19)
Fe1—C3—H3	126.4	C19—C20—H20	126.3
C5—C4—C3	107.9 (3)	C16—C20—H20	126.3
C5—C4—Fe1	69.68 (17)	Fe1—C20—H20	125.3
C3—C4—Fe1	69.48 (17)	H1A—O1—H1B	108.3
C5—C4—H4	126.1		
			
C16—Fe1—C1—C5	172.5 (2)	C6—C7—C8—C9	0.9 (5)
C20—Fe1—C1—C5	131.22 (19)	C7—C8—C9—C10	−0.8 (4)
C17—Fe1—C1—C5	−154.7 (5)	C7—C8—C9—N1	179.2 (3)
C3—Fe1—C1—C5	−80.54 (19)	C12—N1—C9—C8	−49.9 (4)
C2—Fe1—C1—C5	−118.1 (2)	C14—N1—C9—C8	131.7 (3)
C18—Fe1—C1—C5	54.2 (4)	C12—N1—C9—C10	130.1 (3)
C4—Fe1—C1—C5	−36.71 (18)	C14—N1—C9—C10	−48.3 (4)
C19—Fe1—C1—C5	88.6 (2)	C8—C9—C10—C11	0.0 (4)
C16—Fe1—C1—C2	−69.4 (2)	N1—C9—C10—C11	180.0 (3)
C20—Fe1—C1—C2	−110.69 (19)	C9—C10—C11—C6	0.8 (4)
C17—Fe1—C1—C2	−36.6 (6)	C7—C6—C11—C10	−0.8 (4)
C3—Fe1—C1—C2	37.55 (18)	C1—C6—C11—C10	176.2 (3)
C5—Fe1—C1—C2	118.1 (2)	C14—N1—C12—N2	−0.4 (3)
C18—Fe1—C1—C2	172.2 (3)	C9—N1—C12—N2	−179.1 (2)
C4—Fe1—C1—C2	81.37 (19)	C13—N2—C12—N1	0.7 (4)
C19—Fe1—C1—C2	−153.36 (18)	C15—N2—C12—N1	176.4 (3)
C16—Fe1—C1—C6	50.3 (3)	C12—N2—C13—C14	−0.6 (4)
C20—Fe1—C1—C6	9.0 (3)	C15—N2—C13—C14	−176.3 (3)
C17—Fe1—C1—C6	83.0 (6)	N2—C13—C14—N1	0.3 (4)
C3—Fe1—C1—C6	157.2 (3)	C12—N1—C14—C13	0.1 (4)
C5—Fe1—C1—C6	−122.3 (3)	C9—N1—C14—C13	178.7 (3)
C2—Fe1—C1—C6	119.7 (3)	C20—Fe1—C16—C17	−118.9 (3)
C18—Fe1—C1—C6	−68.1 (4)	C3—Fe1—C16—C17	84.9 (3)
C4—Fe1—C1—C6	−159.0 (3)	C5—Fe1—C16—C17	−169.0 (6)
C19—Fe1—C1—C6	−33.7 (3)	C2—Fe1—C16—C17	127.7 (2)
C5—C1—C2—C3	0.4 (3)	C18—Fe1—C16—C17	−37.9 (2)
C6—C1—C2—C3	178.1 (3)	C4—Fe1—C16—C17	52.3 (4)
Fe1—C1—C2—C3	−59.1 (2)	C1—Fe1—C16—C17	167.8 (2)
C5—C1—C2—Fe1	59.49 (19)	C19—Fe1—C16—C17	−80.9 (3)
C6—C1—C2—Fe1	−122.8 (3)	C17—Fe1—C16—C20	118.9 (3)
C16—Fe1—C2—C3	−110.7 (2)	C3—Fe1—C16—C20	−156.1 (2)
C20—Fe1—C2—C3	−153.3 (2)	C5—Fe1—C16—C20	−50.1 (8)
C17—Fe1—C2—C3	−71.4 (2)	C2—Fe1—C16—C20	−113.4 (2)
C5—Fe1—C2—C3	81.2 (2)	C18—Fe1—C16—C20	81.0 (2)
C18—Fe1—C2—C3	−46.6 (6)	C4—Fe1—C16—C20	171.2 (3)
C4—Fe1—C2—C3	37.61 (19)	C1—Fe1—C16—C20	−73.3 (3)
C1—Fe1—C2—C3	119.4 (3)	C19—Fe1—C16—C20	38.0 (2)
C19—Fe1—C2—C3	176.4 (3)	C20—C16—C17—C18	−0.2 (4)
C16—Fe1—C2—C1	129.8 (2)	Fe1—C16—C17—C18	59.6 (2)
C20—Fe1—C2—C1	87.3 (2)	C20—C16—C17—Fe1	−59.8 (2)
C17—Fe1—C2—C1	169.2 (2)	C20—Fe1—C17—C16	38.1 (2)
C3—Fe1—C2—C1	−119.4 (3)	C3—Fe1—C17—C16	−110.8 (2)
C5—Fe1—C2—C1	−38.24 (17)	C5—Fe1—C17—C16	175.0 (3)
C18—Fe1—C2—C1	−166.0 (5)	C2—Fe1—C17—C16	−70.6 (3)
C4—Fe1—C2—C1	−81.81 (18)	C18—Fe1—C17—C16	118.9 (3)
C19—Fe1—C2—C1	57.0 (3)	C4—Fe1—C17—C16	−153.3 (2)
C1—C2—C3—C4	−0.2 (3)	C1—Fe1—C17—C16	−41.3 (7)
Fe1—C2—C3—C4	−59.3 (2)	C19—Fe1—C17—C16	81.7 (2)
C1—C2—C3—Fe1	59.1 (2)	C16—Fe1—C17—C18	−118.9 (3)
C16—Fe1—C3—C4	−155.3 (2)	C20—Fe1—C17—C18	−80.8 (2)
C20—Fe1—C3—C4	172.7 (3)	C3—Fe1—C17—C18	130.3 (2)
C17—Fe1—C3—C4	−113.2 (2)	C5—Fe1—C17—C18	56.1 (4)
C5—Fe1—C3—C4	37.54 (19)	C2—Fe1—C17—C18	170.5 (2)
C2—Fe1—C3—C4	119.5 (3)	C4—Fe1—C17—C18	87.8 (2)
C18—Fe1—C3—C4	−74.2 (3)	C1—Fe1—C17—C18	−160.2 (5)
C1—Fe1—C3—C4	81.4 (2)	C19—Fe1—C17—C18	−37.2 (2)
C19—Fe1—C3—C4	−53.2 (6)	C16—C17—C18—C19	0.9 (4)
C16—Fe1—C3—C2	85.2 (2)	Fe1—C17—C18—C19	60.2 (2)
C20—Fe1—C3—C2	53.2 (4)	C16—C17—C18—Fe1	−59.3 (3)
C17—Fe1—C3—C2	127.3 (2)	C16—Fe1—C18—C19	−81.4 (2)
C5—Fe1—C3—C2	−81.93 (19)	C20—Fe1—C18—C19	−37.2 (2)
C18—Fe1—C3—C2	166.3 (2)	C17—Fe1—C18—C19	−119.1 (3)
C4—Fe1—C3—C2	−119.5 (3)	C3—Fe1—C18—C19	172.6 (2)
C1—Fe1—C3—C2	−38.03 (17)	C5—Fe1—C18—C19	87.8 (2)
C19—Fe1—C3—C2	−172.7 (5)	C2—Fe1—C18—C19	−150.3 (5)
C2—C3—C4—C5	−0.1 (3)	C4—Fe1—C18—C19	130.9 (2)
Fe1—C3—C4—C5	−59.3 (2)	C1—Fe1—C18—C19	50.1 (4)
C2—C3—C4—Fe1	59.2 (2)	C16—Fe1—C18—C17	37.6 (2)
C16—Fe1—C4—C5	167.4 (3)	C20—Fe1—C18—C17	81.9 (2)
C20—Fe1—C4—C5	−42.3 (8)	C3—Fe1—C18—C17	−68.4 (3)
C17—Fe1—C4—C5	−157.5 (2)	C5—Fe1—C18—C17	−153.2 (2)
C3—Fe1—C4—C5	119.2 (3)	C2—Fe1—C18—C17	−31.2 (7)
C2—Fe1—C4—C5	81.6 (2)	C4—Fe1—C18—C17	−110.1 (2)
C18—Fe1—C4—C5	−114.1 (2)	C1—Fe1—C18—C17	169.2 (3)
C1—Fe1—C4—C5	37.02 (18)	C19—Fe1—C18—C17	119.1 (3)
C19—Fe1—C4—C5	−74.8 (2)	C17—C18—C19—C20	−1.2 (4)
C16—Fe1—C4—C3	48.1 (4)	Fe1—C18—C19—C20	58.4 (2)
C20—Fe1—C4—C3	−161.6 (7)	C17—C18—C19—Fe1	−59.6 (2)
C17—Fe1—C4—C3	83.3 (2)	C16—Fe1—C19—C18	81.7 (3)
C5—Fe1—C4—C3	−119.2 (3)	C20—Fe1—C19—C18	120.2 (3)
C2—Fe1—C4—C3	−37.66 (18)	C17—Fe1—C19—C18	38.0 (2)
C18—Fe1—C4—C3	126.7 (2)	C3—Fe1—C19—C18	−26.2 (7)
C1—Fe1—C4—C3	−82.2 (2)	C5—Fe1—C19—C18	−111.7 (2)
C19—Fe1—C4—C3	166.0 (2)	C2—Fe1—C19—C18	164.2 (3)
C3—C4—C5—C1	0.4 (3)	C4—Fe1—C19—C18	−69.5 (3)
Fe1—C4—C5—C1	−58.8 (2)	C1—Fe1—C19—C18	−156.1 (2)
C3—C4—C5—Fe1	59.2 (2)	C16—Fe1—C19—C20	−38.5 (2)
C2—C1—C5—C4	−0.5 (3)	C17—Fe1—C19—C20	−82.2 (2)
C6—C1—C5—C4	−178.1 (3)	C3—Fe1—C19—C20	−146.3 (5)
Fe1—C1—C5—C4	58.8 (2)	C5—Fe1—C19—C20	128.2 (2)
C2—C1—C5—Fe1	−59.30 (19)	C2—Fe1—C19—C20	44.0 (4)
C6—C1—C5—Fe1	123.1 (3)	C18—Fe1—C19—C20	−120.2 (3)
C16—Fe1—C5—C4	−149.1 (6)	C4—Fe1—C19—C20	170.4 (2)
C20—Fe1—C5—C4	169.7 (2)	C1—Fe1—C19—C20	83.7 (2)
C17—Fe1—C5—C4	46.1 (4)	C18—C19—C20—C16	1.1 (4)
C3—Fe1—C5—C4	−37.85 (19)	Fe1—C19—C20—C16	59.9 (2)
C2—Fe1—C5—C4	−81.9 (2)	C18—C19—C20—Fe1	−58.8 (2)
C18—Fe1—C5—C4	84.6 (2)	C17—C16—C20—C19	−0.5 (4)
C1—Fe1—C5—C4	−120.7 (3)	Fe1—C16—C20—C19	−60.7 (2)
C19—Fe1—C5—C4	127.8 (2)	C17—C16—C20—Fe1	60.1 (2)
C16—Fe1—C5—C1	−28.4 (7)	C16—Fe1—C20—C19	118.2 (3)
C20—Fe1—C5—C1	−69.6 (2)	C17—Fe1—C20—C19	80.6 (2)
C17—Fe1—C5—C1	166.8 (3)	C3—Fe1—C20—C19	165.1 (3)
C3—Fe1—C5—C1	82.85 (19)	C5—Fe1—C20—C19	−74.4 (3)
C2—Fe1—C5—C1	38.79 (17)	C2—Fe1—C20—C19	−158.7 (2)
C18—Fe1—C5—C1	−154.69 (19)	C18—Fe1—C20—C19	36.6 (2)
C4—Fe1—C5—C1	120.7 (3)	C4—Fe1—C20—C19	−39.3 (8)
C19—Fe1—C5—C1	−111.49 (19)	C1—Fe1—C20—C19	−114.6 (2)
C5—C1—C6—C11	16.5 (4)	C17—Fe1—C20—C16	−37.6 (2)
C2—C1—C6—C11	−160.7 (3)	C3—Fe1—C20—C16	47.0 (4)
Fe1—C1—C6—C11	108.9 (3)	C5—Fe1—C20—C16	167.5 (2)
C5—C1—C6—C7	−166.6 (3)	C2—Fe1—C20—C16	83.1 (3)
C2—C1—C6—C7	16.2 (4)	C18—Fe1—C20—C16	−81.5 (3)
Fe1—C1—C6—C7	−74.2 (4)	C4—Fe1—C20—C16	−157.5 (7)
C11—C6—C7—C8	−0.1 (4)	C1—Fe1—C20—C16	127.3 (2)
C1—C6—C7—C8	−177.1 (3)	C19—Fe1—C20—C16	−118.2 (3)

### Hydrogen-bond geometry (Å, °)

**Table:**

*D*—H···*A*	*D*—H	H···*A*	*D*···*A*	*D*—H···*A*
O1—H1A···I1	0.84	2.74	3.575 (3)	176
O1—H1B···I1^i^	0.84	2.78	3.616 (3)	176
C12—H12A···O1	0.95	2.39	3.277 (4)	156
C13—H13···I1^ii^	0.95	3.01	3.931 (3)	163

Symmetry codes: (i) −*x*, −*y*+2, −*z*; (ii) *x*, −*y*+3/2, *z*−1/2.

### Table 2 Dihedral angles (°) between the planes.

Note: planes A consists of atoms C1–C5, plane B of atoms C6–C11 and plane C of atoms N1/C12/N2/C13/C14.

**Table:**

Plane	Angle
A and B	17.27 (17)
B and C	48.81 (17)
A and C	32.53 (19)
